# ACE gene insertion/deletion polymorphism has a mild influence on the acute development of left ventricular dysfunction in patients with ST elevation myocardial infarction treated with primary PCI

**DOI:** 10.1186/1471-2261-10-60

**Published:** 2010-12-17

**Authors:** Jiri Parenica, Monika Pavkova Goldbergova, Petr Kala, Jiri Jarkovsky, Martin Poloczek, Jan Manousek, Krystyna Prymusova, Lenka Kubkova, Daniela Tomcikova, Ondrej Toman, Martin Tesak, Josef Tomandl, Anna Vasku, Jindrich Spinar

**Affiliations:** 1 Cardiology Department, Faculty Hospital Brno, Jihlavska 20, Brno 625 00, Czech Republic; 2 Institut of Pathophysiology, Medical Faculty, Masaryk University, Kamenice 753/5, Brno 625 00, Czech Republic; 3 Institute of Biostatistics and Analyses, Masaryk University, Kamenice 126/3, Brno 625 00, Czech Republic; 4 Institut of Biochemisty, Medical Faculty, Masaryk University, Kamenice 753/5, Brno 625 00, Czech Republic

## Abstract

**Background:**

We evaluated the associations among angiotensin-converting enzyme (ACE) gene insertion/deletion (I/D) polymorphism, ACE activity and post-myocardial infarction (MI) left ventricular dysfunction and acute heart failure (AHF) early after presentation with MI with ST-segment elevation (STEMI).

**Methods:**

A total of 556 patients with STEMI treated by primary PCI (421 patients without AHF and 135 patients with AHF) were the study population. The activity of BNP, NT-ProBNP and ACE were measured at hospital admission and 24 h after MI onset. Left ventricular angiography was done before PCI; echocardiography was undertaken between the third and fifth day after MI.

**Results:**

In comparison with the II genotypes group, the DD/ID group had a higher level of ACE activity upon hospital admission (p < 0.001). We found a significantly higher level of ACE activity in patients with moderate LV dysfunction (EF 40-54%) in comparison both with patients with preserved LV function (EF ≥55%) and with patients with severe LV dysfunction (p = 0.028). A non-significant trend towards a higher incidence of mild AHF (22.1% vs. 16.02%, p = 0,093), a significantly higher value of end-systolic volume (ESV/BSA) (30.0 ± 12.3 vs. 28.5 ± 13.0; p < 0.05) and lower EF (50.2 ± 11.1 vs. 52.7 ± 11.7; p < 0.05) in the DD/ID genotypes group was noted. Even after multiple adjustments according to multivariate models, the EF for the DD/ID group remained significantly lower (p = 0,033). The DD/ID genotypes were associated with a significantly higher risk of EF <45% (OR 2.04 [95% CI 1.28; 3.25]).

**Conclusions:**

These results suggest that the I/D polymorphism of ACE is associated with the development of LV dysfunction in the acute phase after STEMI. We demonstrated for the first time an association of the low ACE activity with the severe LV dysfunction, although patients with moderate LV dysfunction had higher level ACE activity than patients with preserved LV function.

## Background

The angiotensin-converting enzyme (ACE) is a membrane-bound, zinc metalloendopeptidase involved in the metabolism of many small peptides, particularly in activating angiotensin I to angiotensin II and inactivating bradykinin via the kallikrein-kininogen system. The enzyme is expressed on the surface of cells in many tissues, and its richest source is the lung. ACE also circulates in a soluble form produced by proteolytic cleavage of the extracellular portion from endothelial cells. Angiotensin II increases contraction of vascular smooth muscle cells. It also affects proliferation of smooth muscle cells; adhesion and diapedesis of monocytes through stimulation of expression of several adhesion molecules and chemoattractants; and the adhesion and aggregation of platelets. Angiotensin II stimulates inflammatory/fibrogenic responses during MI, thus promoting scar formation. On the other hand Angiotensin II-induced reactive oxygen species production may further damage myocardium in the border zones and enlarge infarct size [1]. The ACE gene is located on chromosome 17q23. In the intron 16 of the ACE gene is an I/D polymorphism which contains an insertion (I) and deletion (D)--the presence or absence of 287 bp Alu repeat [2,3]. Homozygotes for the deletion allele (DD) have serum ACE levels higher (on average) as those homozygous for the insertion allele (II), whereas heterozygotes (ID) have intermediate levels [2-4]. The genetic effect accounts for 28%-47% of the total variance of serum ACE [2,3]. Also cardiac ACE activity is higher in subjects with the DD genotype [4]. Early treatment by ACE inhibitors after myocardial infarction (MI) decreases adverse remodeling of the left ventricle, the incidence of heart failure, and mortality [5-11]. Several smaller studies found an increased risk of left ventricular enlargement or remodeling among individuals with the DD/ID genotype after MI [12-16], others were negative, but in the presence of ACEI therapy [17].

However recently published results detected new single-nucleotid polymorphisms associated with low mRNA expression and with adverse cardiovascular outcomes [18].

Based on these results, we hypothesized a higher activity of ACE in the acute stage of ST segment elevation myocardial infarction (STEMI) in patients with DD/ID genotypes compared with individuals homozygous for the insertion allele (II). We investigated if the ACE I/D polymorphism and levels of ACE activity in the acute period after STEMI treated with primary PCI were associated with post-MI left ventricular dysfunction and evidence of acute heart failure.

## Methods

### Study population

Written informed consent was obtained from all subjects before participation in the study. The study protocol complied with the Declaration of Helsinki, and was approved by the local Ethics Committee of Faculty Hospital Brno and by the Ethics Committee of the Masaryk University in Brno (Brno, Czech Republic).

From November 2005 to October 2008, 913 patients with STEMI were referred for primary percutaneous coronary intervention (PCI). They were admitted to the Coronary Care Unit (CCU) of Internal Cardiological Department of Faculty Hospital Brno. The diagnosis of STEMI was based on symptoms consistent with MI in conjuction with appropriate changes on electrocardiography (ECG) (ST-segment elevation or new left bundle branch block (LBBB)) and elevation in the levels of markers of myocardial necrosis (troponin I). Time from the onset of chest pain until primary PCI was <12 h. Standard therapy, including ACE inhibitors, beta-blockers and statins, was given as soon as possible after primary PCI. Exclusion criteria were: age >80 years (n = 112); known or newly diagnosed malignancy; inflammatory diesease or connective-tissue diesease; known previous LV dysfunction; estimated life expectancy <12 months (n = 48); refusal to sign the consent inform or non-compliance (n = 74); geographic reasons (distance from the place of residence to the hospital of >100 km without the possibility of follow-up; n = 35). In total, 51 patients were not included because of technical and organizational problems. Genotypes were not obtained for 37 patients. In total, 556 consecutive patients with STEMI were included in the present study. A control population of 200 healthy subjects (age, 48-80 years; median, 65 years; 76.5% male) without a history of CAD or heart failure were evaluated by specialized cardiologists in the Brno region. The diagnosis of acute heart failure was assessed according to clinical signs upon hospital admission and/or during hospitalization (Killip class I-IV). Mild acute heart failure was defined as pulmonary congestion with wet rales in the lower half of the lung fields or S3 gallop. The study population was divided into two groups according to ACE I/D polymorphism (DD/ID group and II group).

### Laboratory methods

Samples of venous blood for analyses of the activity of B-type natriuretic peptide (BNP), N-terminal pro B-type natriuretic peptide (NT-ProBNP) and ACE were drawn immediately upon hospital admission before primary PCI (sample 1). Blood samples were drawn again at 24 h after the onset of chest pain (sample 2). Samples were centrifuged within 20 min in a refrigerated centrifuge, and the plasma and serum stored at -80°C. Standard biochemical and hematological blood tests were done immediately at hospital admission before primary PCI and 24 h after the onset of chest pain (including Troponin-I ADSV, Abbott Laboratories, Abbott Park, USA). BNP was analyzed using the AxSYM BNP-Microparticle Enzyme Immunoassay (Abbott Laboratories, Abbott Park, USA). NT-ProBNP was analyzed using the Cobas E411 NT-proBNP Imunoassay Kit (Roche Diagnostics, Indianapolis). The activity of ACE was analysed using ACE kinetic assay (Bühlmann laboratories AG, Switzerland).

### Echocardiography assessment

Echocardiography was carried out during the index admission (between the third and fifth day after MI onset). Left ventricular end-systolic volume (LVESV), left ventricular end-diastolic volume (LVEDV), and left ventricular ejection fraction (LVEF) were estimated using the bi-planar Simpson's rule from apical two- and four-chamber views (93.8% and 94.6% of all measurements in DD/ID genotype group, resp. in II genotype group) or by the Teicholz formula. Echocardiography was assessed by two operators using Vivid 7 or Vivid i (GE Vingmed Ultrasound).

### Invasive measurement

Ejection fraction (EF) was determined by LV angiography (5-F pigtail catheter) before primary PCI. A diseased coronary vessel was defined as having ≥50% reduction of the intraluminal diameter of any of the coronary arteries (left main, left anterior descending, left circumflex, right coronary) or the main branches with a diameter ≥2.0 mm. Significant left main artery stenosis was classified as "two-vessel disease".

### Genetic analysis

DNA was extracted from peripheral leukocytes using the standard proteinase K technique. Genotyping of the insertion/deletion polymorphism in angiotensin I-converting enzyme (I/D ACE) was done using polymerase chain reaction (PCR) methods according to the method of Rigat and Shanmugam [19]. DNA amplification was undertaken in a volume of 25 μL containing 50 ng genomic DNA with 1 unit Taq polymerase (MBI Ferments, USA). Using gel electrophoresis, PCR products were distinguished at 1.5% Serva with ethidium bromide in UV light as a 190 bp fragment in the absence of an Alu repeat insertion and a 490 bp fragment in the presence of the insertion (genotypes described as II-490 bp, ID-490+190 bp, and DD-190 bp). Blind samples were used to control the reliability of genotyping.

### Statistical analyses

Summary statistics for categorical variables are shown as frequencies. Normally distributed continuous variables are shown as mean and SD or median and 5-95^th ^percentile range for variables with non-normal distribution. Within all groups, the distributions of genotypes and consistency of genotype frequencies with the Hardy-Weinberg equilibrium were tested using the χ^2 ^test on a contingency table of observed versus predicted genotype frequencies. The allelic frequences were compared by Fisher's exact test. The point estimates of the risk, the odds ratio (OR) and 95% confidence interval (CI) based on logistic regression were estimated and supplemented by their significance (Ward's test). Changes within groups were analyzed by Wilcoxon signed-ranks test, and differences between two groups were analyzed by the Mann-Whitney U test. Correlations were analyzed by Spearman's rank order correlation coefficient. A probability value of α < 0.05 was considered to indicate significance. Multivariate linear regression was applied for adjustment of markers of LV dysfunction on the influence of other variables. Preselection of variables based on univariate linear regression and a backward stepwise algorithm were used for the definition of these models. The power of tests and sample size were evaluated by EpiInfo6. Statistical analyses were undertaken using Stat View version 8.0 (SAS Institute Incorporated, 2008), SPSS 18.0.3 (IBM, Corporation 2010) and STATISTICA 8.0 (StatSoft, Inc. 2008).

## Results

Genotypes were obtained for 556 patients of the study group. Demographic characteristics and the clinical background of patients are shown in Table 1. All patients and control subjects were Caucasian. Patients with DD/ID genotypes were more often diabetic (p < 0.05) with higher stress glucose level during admission (p < 0.05). Previous treatment (Table 1), treatment in the course of hospitalization and at the time of hospital discharge with ACE inhibitors, beta-blockers and statins were comparable between genotype groups (ACE inhibitors and/or AT II blockers 94.7%, beta-blockers 96.6%, statins 96.3%). The median time from the onset of chest pain to admission to the CCU was 170 min and the median "door-to-balloon" time was 63 min. The times were comparable between genotype groups (p = NS).

**Table 1:** Baseline characteristics of patients, treatment, and neurohumoral measurements in the ACE genotype group

	All patients	DD/ID genotype	II genotype	p*
	N = 556	N = 393	N = 163	DD/ID vs. II
Age (years)	62.7 ± 10.5	62.7 ± 10.3	62.6 ± 10.8	0.972
Males	74.6	75.1	73.6	0.722
Weight (kg)	83.9 ± 14.0	84.2 ± 14.1	83.1 ± 13.9	0.330
BMI (kg/m^2^)	28.1 ± 3.9	28.1 ± 4.0	27.9 ± 3.7	0.893
BP systolic (mmHg)	135.6 ± 27.0	135.3 ± 27.2	136.1 ± 26.5	0.315
BP diastolic (mmHg)	74.7 ± 14.4	74.5 ± 14.2	75.1 ± 15.0	0.302
Heart rate (per min)	73.0 (52.0-108.0)	73.0 (52.0; 107.6)	72.0 (50.4; 109.4)	0.941
Time delay (min)	238.0 (123.0-650.0)	245.0 (122.1; 667.0)	228.0 (121.6; 590.2)	0.282
AHF	27.0	28.2	23.9	0.296
Mild AHF (%)	20.3	22.1	16.0	0.093
Smokers^† ^(%)	54.7	54.7	54. 6	0.982
Diabetes (%)	27.9	30.5	21.5	**0.027**
Hypertension (%)	57.4	58.3	55.2	0.508
ACE inhibitors (%)	25.4	24.4	27.6	0.435
AT II (%)	7.0	7.4	6.1	0.597
Beta-blockers (%)	27.2	27.7	25.8	0.634
Statins (%)	16.5	16.5	16.6	0.994
Glucose (mmol/L)	8.1 (5.5-17.3)	8.2 (5.7; 17.4)	7.7 (5.3; 16.7)	**0.006**
Troponin I (ng/mL)	50.2 (3.1-182.1)	48.9 (3.2; 186.5)	52.2 (2.4; 181.3)	0.940
BNP at hospital admission (pg/mL)	66.3 (15.0-489.7)	66.2 (15.0; 504.1)	68.0 (15.0; 491.0)	0.314
BNP after 24 h (pg/ml)	275.2 (68.2-890.0)	286.7 (68.1; 892.9)	269.3 (70.0; 1145.5)	0.955
NT-ProBNP at hospital admission (pg/mL)	223.0 (33.0-4 233.0)	230.0 (33.0; 4512.0)	183.0 (28.1; 5350.2)	0.435
NT-ProBNP after 24 h (pg/mL)	1946.0 (370.0-11 542.0)	2019.0 (384.1; 10197.9)	1805.0 (355.4; 15008.4)	0.432
ACE activity (IU/L)	30.6 (4.1-68.3)	33.6 (4.1; 70.6)	24.1 (4.4; 63.8)	**< 0.001**

Continuous variables are presented as mean, SD, median, 5-95 percentiles, and categorized as percentage proportion. * The Mann-Whitney U test or chi-square test were used for testing differencies between genotype groups ^† ^smokers = current smokers and ex-smokers; BMI - Body mass index; Time delay = time between onset of chest pain and ballon dilatation

Significant differences were not observed in the distributions of ACE genotypes and/or allele frequences between patients with acute MI (DD 29.5%, ID 41.2%, II 29.3%; I 46.5%, D 53.5%) and healthy controls (DD 28.1%, ID 50.7%, II 21.2%; D 50.1%, I 49.9%) (p = NS).

In comparison with the II genotype group, the DD genotype group had a higher level of ACE activity upon admission, while ID genotype group had an intermediate level of ACE activity (medians of ACE activities of DD vs ID vs II genotype groups were 36.7 U/L vs 31.4 U/L vs 24.1 U/L, p < 0.001). We found a significantly higher level of ACE activity for patients with moderate LV dysfunction (EF 40-54%; n = 301; described by median and 5-95^th ^percentile (30.9 U/L (10.2-65.5)) in comparison both with patients with preserved LV function (EF ≥55%; n = 185; 28.5 U/L (8.2-60.4)) and in patients with severe LV dysfunction (EF ≤40%; n = 70; level of ACE activity 27.8 U/L (5.4-62.6)) (p = 0.028). Using multivariate linear regression, only age, previous treatment with ACE inhibitors or AT1-antagonists and I/D polymorphism were detected as variables with a statistically significant influence on ACE activity (25% of variability of ACE activity was explained by a given model; 4.9% of the total variability of ACE activity could be explained by ACE gene I/D polymorphism in this model).

Total hospital mortality was 3.54% with no difference among groups (DD 3.65%, ID 3.06%, II 4.87%). In total, 27.0% of patients had signs and symptoms of acute heart failure AHF (Killip class II and above) during hospitalization. We found a non-significant trend towards a higher incidence of mild AHF for the DD/ID genotypes group (Table 1). Hemodynamic and echocardiographic measurements are shown in Table 2. In the case of DD/ID genotypes carriers, we found a significantly higher value of ESV/BSA and significantly lower EF. We created a multivariate model and we found that EF is dependent only on the previous myocardial infarction, creatinine, infarct related artery (RIA), number of affected arteries and the time from the oncet of chest pain till the patency of infarct-related artery (Table 3). The EF and other parameters of LV dysfunction were multiple adjusted according to the multivariate models (Table 3) and the values were compared between DD/ID and II genotypes carriers. Only the adjusted EF for DD/ID group was still significantly lower (adjusted EF described by median and 5-95^th ^percentile for DD/ID genotype group 50.5% (49.5; 51.4) vs II genotype group 52.0% (50.4; 53.6) (p = 0.033)). The DD/ID group had a significantly higher risk of EF <45% before hospital discharge (OR (95% CI) 2.04 (1.28; 3.25) (p = 0.003)), a similar result was found for DD/ID subgroup of patients without diabetes mellitus (OR (95% CI) 2.17 (1.25; 3.80) (p = 0.006).

**Table 2:** Invasive and echocardiographic measurements

	All patients	DD/ID genotype	II genotype	p*
	N = 556	N = 393	N = 163	DD/ID vs. II
DCV	1.89 ± 0.80	1.92 ± 0.80	1.83 ± 0.80	0.236
IRA - LM	2 (0.4%)	2 (0.5%)	0	0.893
IRA - LAD (%)	256 (46.0%)	183 (46.6%)	73 (44.8%)	0.772
IRA - RCx (%)	74 (13.3%)	51 (13.0%)	23 (14.1%)	0.825
IRA - RCA (%)	224 (40.3%)	157 (39.9%)	67 (41.1%)	0.875
Initial TIMI				
0-1	380 (68.3%)	270 (68.7%)	110 (67.5%)	0.050
2	91 (16.4%)	71 (18.1%)	20 (12.3%)	
3	85 (15.3%)	52 (13.2%)	33 (20.2%)	
TIMI after PCI				
0-1	13 (2.3%)	10 (2.5%)	3 (1.8%)	0.830
2	51 (9.2%)	37 (9.4%)	14 (8.6%)	
3	492 (88.5%)	346 (88.0%)	146 (89.6%)	
EF (angiography)	49.4 ± 12.2	49.3 ± 21.4	49.7 ± 12.0	0.644
EDV/BSA (mL/m^2^)	62.4 ± 17.0	62.1 ± 16.9	63.2 ± 17.5	0.583
ESV/BSA (mL/m^2^)	29.6 ± 12.5	30.0 ± 12.3	28.5 ± 13.0	**0.049**
EF (echo) (%)	50.9 ± 11.3	50.2 ± 11.1	52.7 ± 11.7	**0.006**

Continuous variables are presented as mean, SD, median, 5-95 percentiles, and categorized as percentage proportion. *The Mann-Whitney U test or chi-square test were used for testing differencies between genotype groups

DCV: Diseased coronary vessels; IRA: infarct-related artery; LM: left main; LAD: left anterior descending coronary artery; RCx: ramus cirumflexus; RCA: right coronary artery; EF: ejection fraction of the left ventricle; EDV: end-diastolic volume; ESV: end-systolic volume; BSA: Body surface area.

**Table 3:** Multivariate models for adjusting of parameters of left ventricle dysfunction

	Ejection fraction (echo)	Ejection fraction (angiography)	EDV/BSA	ESV/BSA1	BNP1	BNP2	NT-pro BNP1	NT-pro BNP2
Age	-	-	-	-	< 0.001	< 0.001	< 0.001	< 0.001
Sex (Women)	-	-	0.001	0.003	0.004	< 0.001	< 0.001	< 0.001
BMI	-	-	-	-	-	-	0.017	-
Diabetes mellitus	0.336	-	0.140	0.067	-	-	-	-
Previous instability	-	-	-	-	0.037	-	< 0.001	0.001
Infarction	< 0.001	0.001	< 0.001	< 0.001	-	-	-	-
Betablocators	-	-	-	-	< 0.001	-	0.002	-
Creatinine3	0.004	< 0.001	-	-	< 0.001	< 0.001	< 0.001	< 0.001
IRA - RIA	< 0.001	< 0.001	-	0.010	-	< 0.001	-	< 0.001
DCV	0.001	-	-	0.007	0.260	0.005	0.011	0.002
Time delay (min)	0.003	< 0.001	-	-	< 0.001	0.069	< 0.001	0.002

R^2^	17.3%	18.6%	5.7%	9.0%	26.9%	31.6%	42.0%	42.7%

Statistical significance of relationship was analyzed by means of linear regression; parameters ESV/BSA, BNP and NT-proBNP were log transformed prior to analysis. R^2 ^stands for variability explained by given model.

EDV: end-diastolic volume; ESV: end-systolic volume; BSA: Body surface area; BMI: Body mass index; IRA: infarct-related artery; DCV: Diseased coronary vessels

## Discussion

### ACE gene polymorphism and prognosis after MI

Samani [20] concluded that I/D polymorphism did not significantly influence the risk of mortality after MI in a group of 684 patients with MI. Yoshida [21] revealed that the endpoints of the study (cardiac death, reccurent MI or unstable angina) were significantly associated with the ACE ID polymorphism (RR = 4.49), and that the DD/ID genotype was associated with a higher occurrence of the endpoints. Similar results were revealed by Palmer [12]. In a study of 978 patients after MI, the DD/ID genotypes were associated with a higher risk of mortality with an OR of 8.03 (95% CI, 2.16-29.88; p < 0.05). In the present study, an association of in-hospital mortality and I/D ACE polymorphism was not observed. This was probably because of the very short duration of follow-up (this work explored only the time of hospitalization) and fewer subjects.

### ACE gene polymorphism, the level of ACE activity and left ventricle function

Circulating ACE probably originates from vascular endothelial cells. Higher levels of plasma ACE were found for the DD/ID genotypes of healthy subjects [3] as well as of patients with CAD [22,23]. At the same time Danser [4] explored ACE activity in the cardiac tissue of subjects who died of non-cardiac disorders, and the highest levels were in subjects with the DD genotype. An elevated activity of ACE during the acute phase of STEMI in association with I/D polymorphism ACE has not been documented. We demonstrated for the first time a clear association of a higher activity of ACE in DD genotype group in comparison with II group in acute phase of STEMI, while the ID genotype group had an intermediate level of ACE activity. There is a lack of data concerning the ACE activity and its interaction with AHF and LV dysfunction. Cambien [22] indicated that a higher level of ACE in plasma could be a risk factor for MI independent of the I/D polymorphism. Nakai [24] concluded that the deletion polymorphism of the ACE gene was associated with a higher activity of ACE in serum and carried an increased risk of CAD in Japanese subjects. Other results were presented by Gardemann [23], who concluded that increased ACE activity was not a risk factor for CAD or MI. A completely new look Johnson [18] brought at 2009, he detected three promotor single-nucleotide polymorphisms (SNPs) of the ACE gene connected with reduced ACE mRNA expression in cardiac tissue. These identified SNPs (rs7213516 and rs4290) were tested in a large cohort of 1032 hypertonics and they were associated with adverse cardiovascular outcomes, largely attributable to nonfatal MI in African Americans. The high allele frequency was found in African Americans (16%), but low in Hispanics (4%) and very low in Caucasians (< 1%). We do not suppose these SNPs play a relevant role in our study population according to low allele frequency in Caucasians only 1 or 2 subjects could be expected. But very important is an idea that very low level of ACE activity could be harmfull. So far the higher levels of ACE activity associated with DD/ID genotypes of ACE were connected with worse prognosis [24], but existing results were not consistent [23,24]. For the first time our results suggest a non-linear type of a dependence of ACE activity and adverse cardiac outcomes. As we showed, higher levels of ACE activity were associated with moderate LV dysfunction (EF 40-54%), but lower levels of ACE activity were found in patients with preserved LV function (EF ≥ 55%) as well as in patients with severe LV dysfunction (EF < 40%). The results of Johnson study [18] and the Valsartan Heart Failure (Val-HeFT) trial [25] raise the possibility, that excessive neurohormonal inhibition may contribute to adverse outcomes in heart failure treatment.

### ACE gene polymorphism and LV systolic dysfunction after MI

Infarct size is one of the most important determinants of the progression of the LVEDV [26]. Pinto et al. [16] reported that enlargement of the left ventricle 12 months after an anterior MI was more pronounced in patients with the DD genotype than in patients with the ID or II genotypes. They did not find any differences in the volumes of the left ventricle in the acute stage of MI [16]. Ohmichi [15] associated the DD and ID genotypes of the ACE gene with the progression of the left ventricular end-diastolic volume index (LVEDVI) and end-systolic volume index (LVESVI) 4 months after MI. Multiple regression analysis revealed that the LVESVI at echocardiography 7 ± 4 days after MI and ACE I/D polymorphism are predictors of the increases in the LVEDVI a LVESVI on the second echocardiogram. In a group of 43 patients with old anteroseptal MI-treated PCI (they were not treated by ACE inhibitors), Nagashi [14] found a significantly higher LV end-systolic and end-diastolic dimension in the deletion group. Ulgen [13] proved the relationship of ACE gene polymorphism and LV remodeling in the early period in 142 patients with anterior MI. Echocardiographic examinations were undertaken within 24 h of MI and on the fifth day. LV systolic diameters, diastolic diameters and EF between the first and second echocardiographic results were significantly different in the DD group. Palmer [12] demonstrated a significantly greater LVEDV and the increased LVESV bordered on significance when 223 DD individuals were compared with grouped ID ad II patients (n = 572). This was not confirmed in a study by Zee [17], which provided no evidence of an association of the ACE I/D polymorphism with the risk of LV remodeling post-MI in the presence of ACE-I therapy. During the fifth day after MI, we demonstrated a significantly greater LVESVI and worse EF for the DD/ID genotypes in a homogenous group of patients with STEMI despite treatment by primary PCI and full pharmacological treatment (including ACE inhibitors given as soon as possible). We consider the difference of EF between the both genotype groups on the border of clinical significance, the absolute value of the EF difference was only 1.5% after adjustments for other variables. This borderline influence of ACE polymorphism may explain inconsistent results of previous smaller studies on the development of left ventricular dysfunction, especially when ACE inhibitors were administered.

### ACE gene polymorphism and AHF

We did not find data in the literature concerning the clinical signs of AHF during the acute stages of MI and polymorphism of the ACE gene. An accurate assignation of diagnosis of acute heart failure could be influenced in border line by individual assessment of the subject status. We found a non-significant trend for an association of I/D ACE polymorphism and signs of mild AHF.

### ACE gene polymorphism and BNP and NT-proBNP

BNP is a cardiac hormone that is secreted mainly from the ventricles in response to excessive stretching of cardiomyocytes. NT-proBNP is an inactive molecule resulting from the cleavage of the prohormone Pro-BNP. Palmer [12] showed that patients with the DD genotype had a significantly higher plasma level of BNP, N-terminal BNP, and endothelin levels within 96 h after MI than those observed in the II/ID group. These data was in correlation with a greater LVEDV in the DD group. In our study a significant association between DD/ID polymorphism of the ACE gene and BNP or NT-proBNP was not found even after the multiple adjustments for variables significantly influencing the level of BNP or NT-proBNP. Probably the later assesment of BNP/NT-proBNP after MI (96 hours in Palmer study vs 24 hours after MI onset in our study) is likely more relevant to determine subsequent development of left ventricular dysfunction in which the ACE polymorphism may participate.

### Comparison with previous studies

Previous studies have not defined exactly the type of MI (NSTEMI or STEMI) and the manner of revascularization. Just the type and time of revascularization are crucial factors for the dysfunction and remodeling of the left ventricle and congestive heart failure. Primary PCI is associated with a significant reduction in mortality and reinfarction in patients with STEMI, above all in patients with AHF [27,28]. Several trials and studies proved a positive influence of treatment by ACE inhibitors for the progression of LV remodeling and heart failure. Although patients had full treatment of acute coronary syndrome with STEMI--revascularization by primary PCI (PCI was succesful in 97.5% of patients), ACE inhibitors, beta-blockers and statins were usually administered immediately after PCI, we found a border interaction between I/D ACE polymorphism and signs of LV systolic dysfunction up to 5 days after MI. For the first time we confirmed definitely that the level of ACE activity is influenced by I/D polymorphism in acute stage of myocardial infarction, although only small part of ACE activity variability may be explained by this polymorphism. We demonstrated that the higher level of ACE activity is associated with the development of the moderate left ventricular dysfunction in comparison with ACE activity in patients with preserved left ventricular function. However the severe left ventricular dysfunction (EF < 40%) was associated also with low ACE activity. These new results may help explain ambiquous findings of previous studies.

### Limitation of the study

Although our study population belongs to larger cohorts, on which interraction between left ventricular dysfunction and I/D polymorphism was investigated so far, a much larger group would be better to set up. The results brought new information about interraction among I/D gene polymorphism, ACE activity and the development of left ventricular dysfunction. However we do not believe the stratification of patients according to I/D polymorphism and ACE activity is useful in the daily clinical practice.

## Conclusion

The present study clearly demonstrated an association between I/D gene polymorphism and the development of LV dysfunction in the acute stage of STEMI despite revascularization by primary PCI and early treatment using ACE inhibitors and beta-blockers. For the first time we confirmed definitely that the level of ACE activity is influenced by I/D polymorphism also in acute stage of myocardial infarction. We demonstrated that higher level of ACE activity was associated with the development of the moderate left ventricular dysfunction in comparison with ACE activity of patients with preserved left ventricular function, however the severe left ventricular dysfunction (EF < 40%) was associated with low ACE activity as well.

## Competing interests

The authors declare that they have no competing interests.

## Authors' contributions

JP drafted the manuscript and conceived of the study, MGP drafted genetic part of the manuscript, PK participated in the study design, MP interpreted invasive data, JM and MT and KP carried out the echocardiography and interpreted its data, LK helped to draft the manuscript, JJ and DT participated in the design of the study and performed the statistical analysis, OT participated in the design of the study and helped to draft the manuscript, JT carried out the biochemistry analysis and interpreted the data, AV participated in the study design and helped to draft the manuscript, JS participated in the study design and helped to draft the manuscript. All authors have red and approved the final manuscript.

## Pre-publication history

The pre-publication history for this paper can be accessed here:

http://www.biomedcentral.com/1471-2261/10/60/prepub
